# Supplementary material for: Control and regulation of acetate overflow in Escherichia coli
Source: eLife. 2021 Mar 15;10:e63661. doi: 10.7554/eLife.63661 (PMC8021400; doi:10.7554/eLife.63661)
Supplement: Supplementary file 1. [file elife-63661-supp1.zip › acetate_regulation/results/Figure 3.pdf]

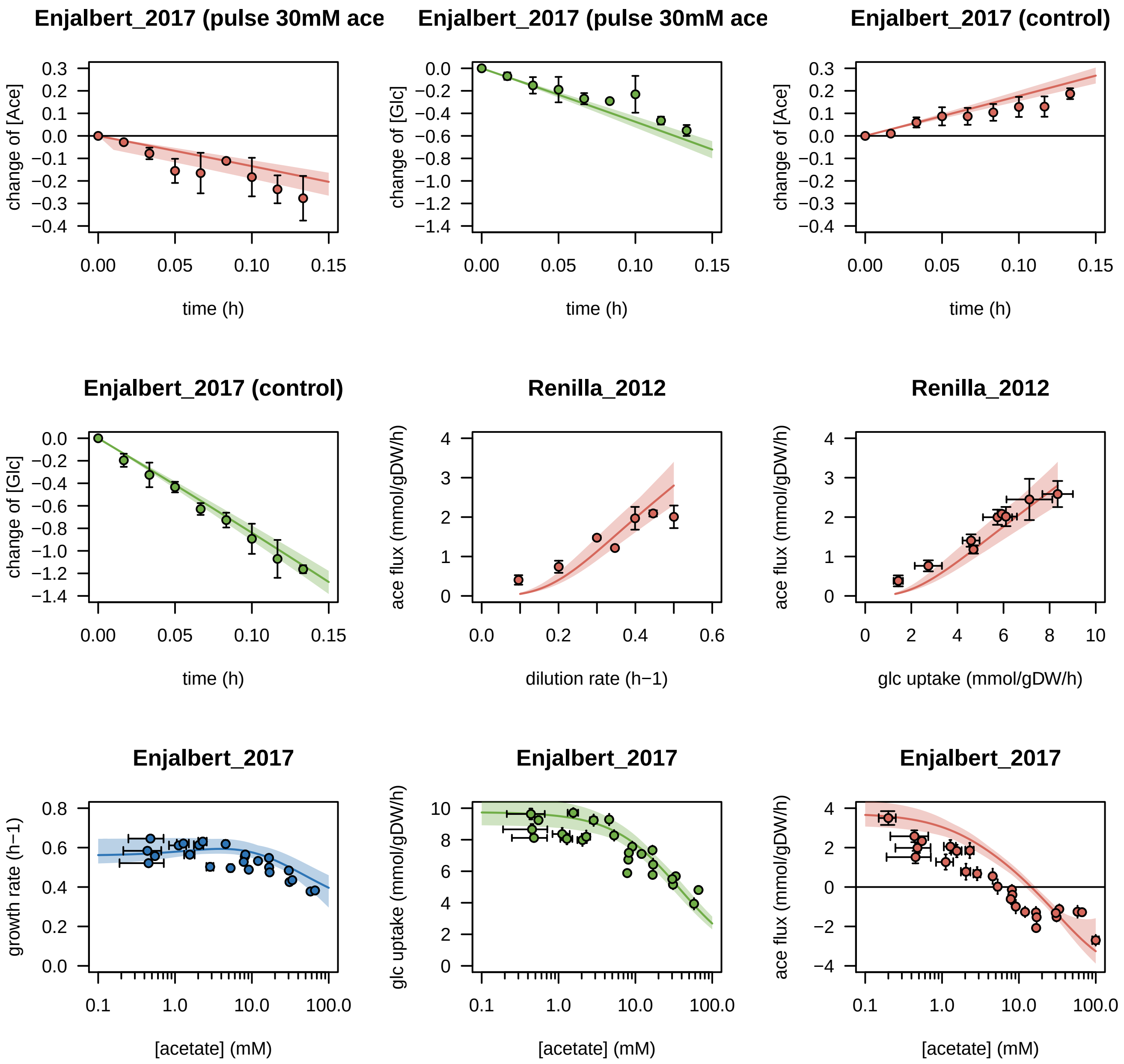
Enjalbert_2017 (pulse 30mM ace
change of [Ace]
time (h)
Enjalbert_2017 (pulse 30mM ace
change of [Glc]
time (h)
Enjalbert_2017 (control)
change of [Ace]
time (h)
Enjalbert_2017 (control)
change of [Glc]
time (h)
Renilla_2012
ace flux (mmol/gDW/h)
dilution rate (h−1)
Renilla_2012
ace flux (mmol/gDW/h)
glc uptake (mmol/gDW/h)
Enjalbert_2017
growth rate (h−1)
[acetate] (mM)
Enjalbert_2017
glc uptake (mmol/gDW/h)
[acetate] (mM)
Enjalbert_2017
ace flux (mmol/gDW/h)
[acetate] (mM)
