# Supplementary material for: Control and regulation of acetate overflow in Escherichia coli
Source: eLife. 2021 Mar 15;10:e63661. doi: 10.7554/eLife.63661 (PMC8021400; doi:10.7554/eLife.63661)
Supplement: Supplementary file 1. [file elife-63661-supp1.zip › acetate_regulation/results/Figure 3–figure supplement 1.pdf]

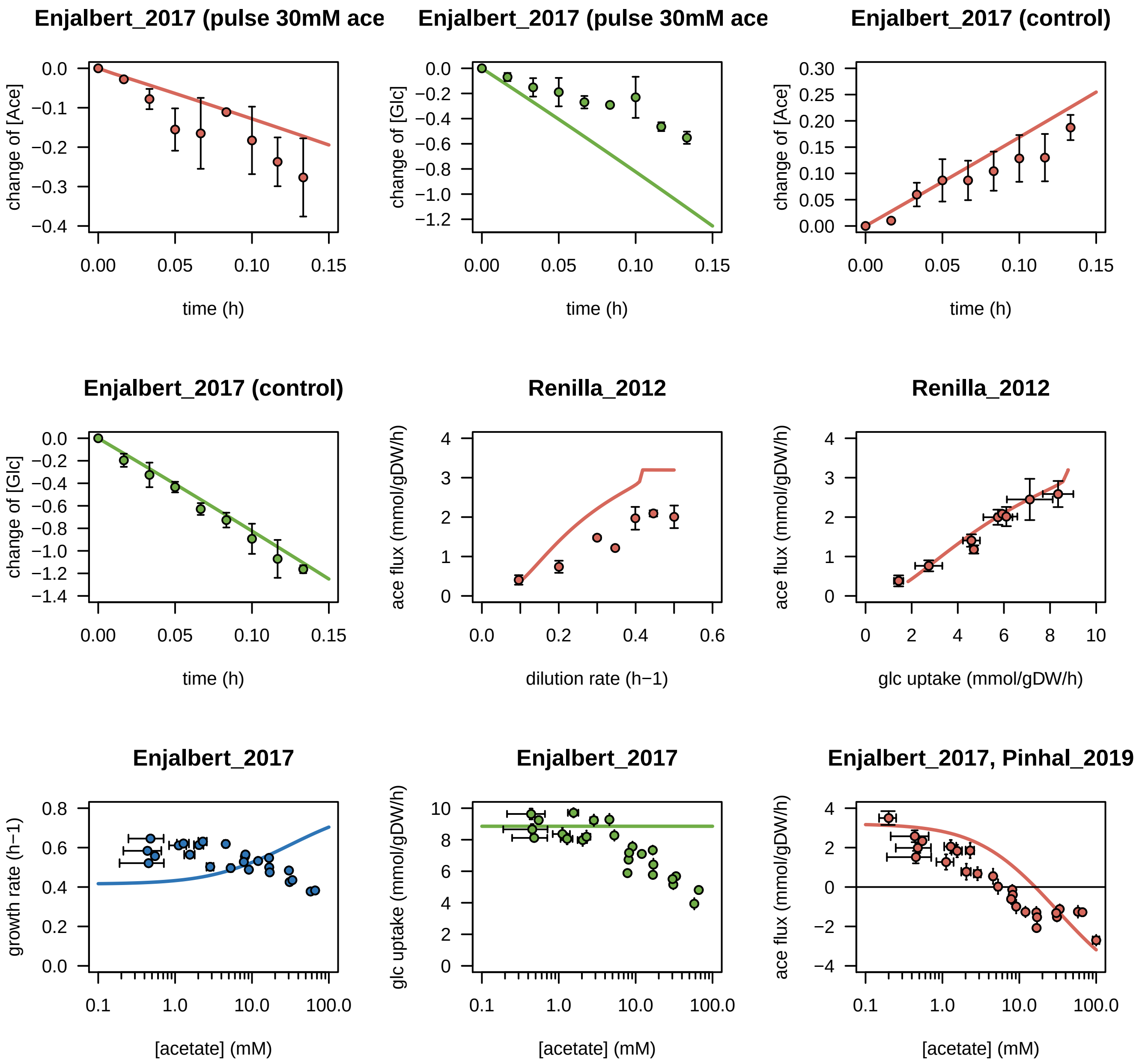
Enjalbert_2017 (pulse 30mM ace
change of [Ace]
0.0
−0.1
−0.2
−0.3
−0.4
0.00
0.05
0.10
0.15
time (h)
Enjalbert_2017 (pulse 30mM ace
change of [Glc]
0.0
−0.2
−0.4
−0.6
−0.8
−1.0
−1.2
0.00
0.05
0.10
0.15
time (h)
Enjalbert_2017 (control)
change of [Ace]
0.30
0.25
0.20
0.15
0.10
0.05
0.00
0.00
0.05
0.10
0.15
time (h)
Enjalbert_2017 (control)
change of [Glc]
0.0
−0.2
−0.4
−0.6
−0.8
−1.0
−1.2
−1.4
0.00
0.05
0.10
0.15
time (h)
Renilla_2012
ace flux (mmol/gDW/h)
4
3
2
1
0
0.0
0.2
0.4
0.6
dilution rate (h−1)
Renilla_2012
ace flux (mmol/gDW/h)
4
3
2
1
0
0
2
4
6
8
10
glc uptake (mmol/gDW/h)
Enjalbert_2017
growth rate (h−1)
0.8
0.6
0.4
0.2
0.0
0.1
1.0
10.0
100.0
[acetate] (mM)
Enjalbert_2017
glc uptake (mmol/gDW/h)
10
8
6
4
2
0
0.1
1.0
10.0
100.0
[acetate] (mM)
Enjalbert_2017, Pinhal_2019
ace flux (mmol/gDW/h)
4
2
0
−2
−4
0.1
1.0
10.0
100.0
[acetate] (mM)
