# Supplementary figures and images for: Control and regulation of acetate overflow in Escherichia coli
Source: eLife. 2021 Mar 15;10:e63661. doi: 10.7554/eLife.63661 (PMC8021400; doi:10.7554/eLife.63661)

[acetate]=1mM

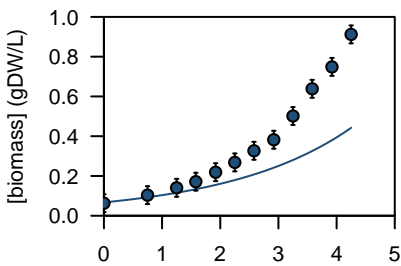

[acetate]=10mM

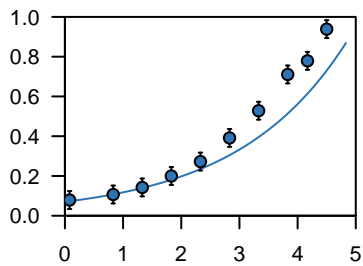

[acetate]=30mM

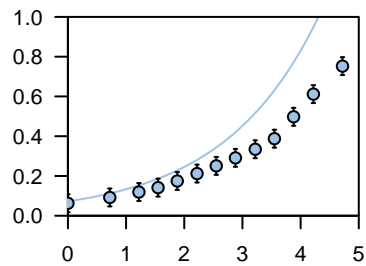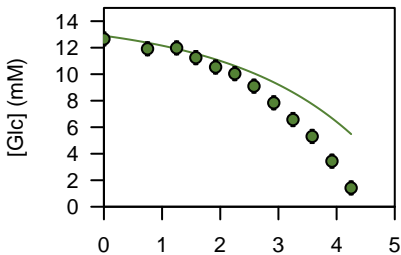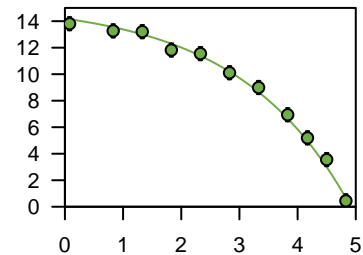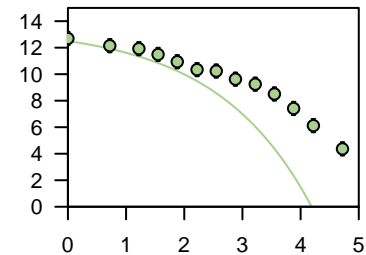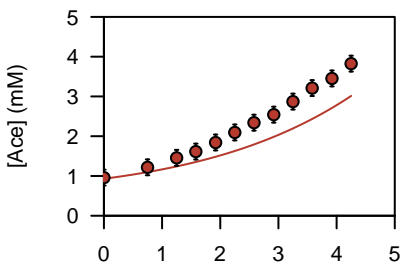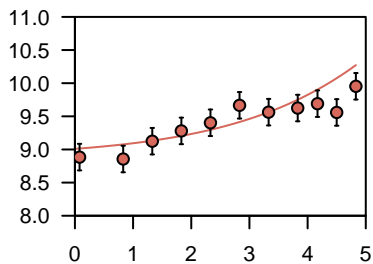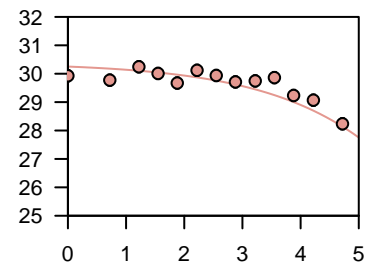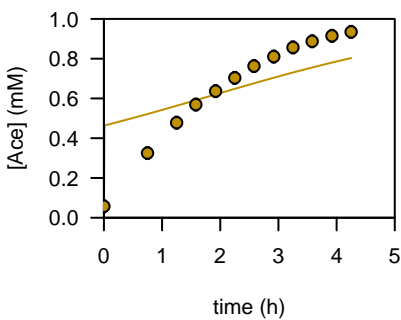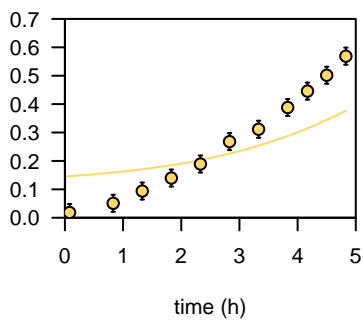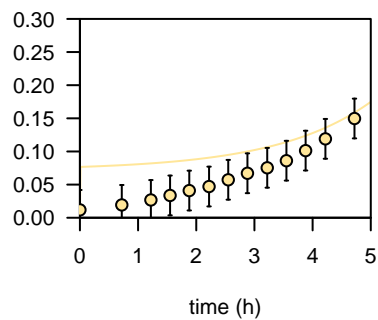

Supplement: Supplementary file 1. [file elife-63661-supp1.zip › acetate_regulation/results/Figure 1-figure supplement 1.pdf]

[acetate]=1mM

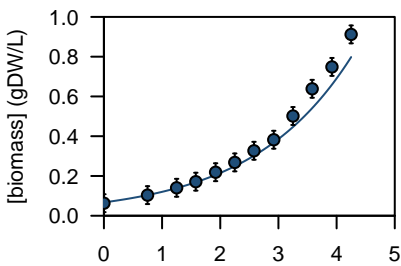

[acetate]=10mM

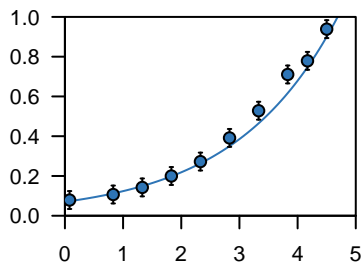

[acetate]=30mM

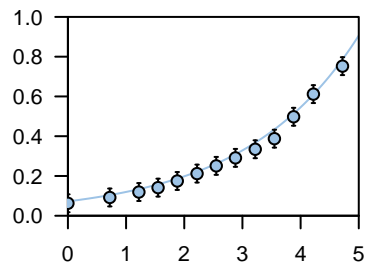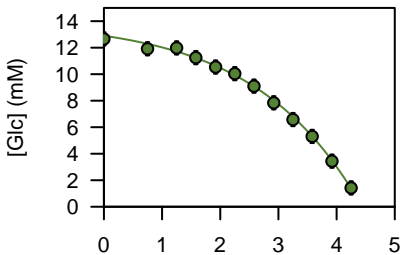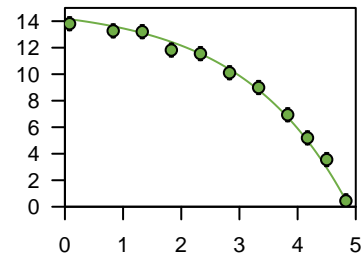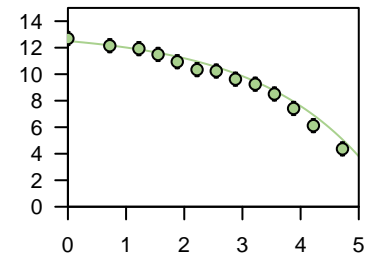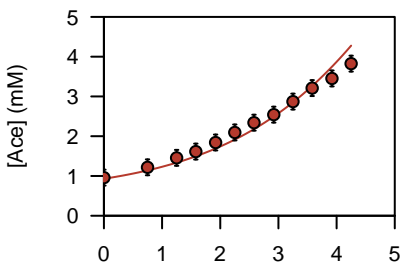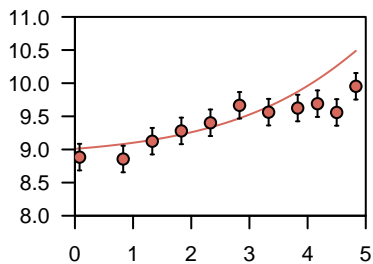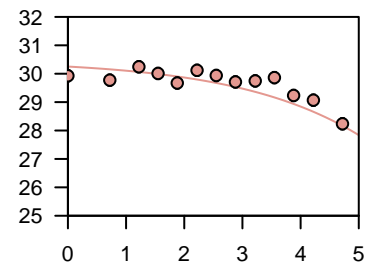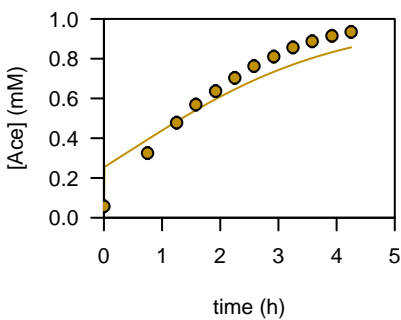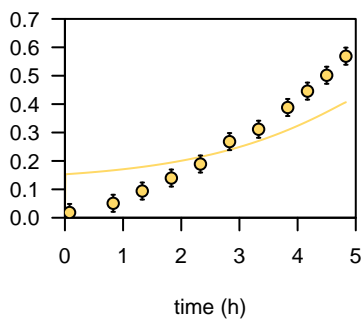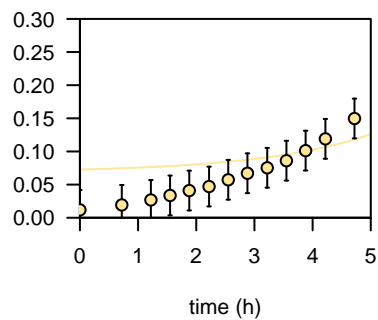

Supplement: Supplementary file 1. [file elife-63661-supp1.zip › acetate_regulation/results/Figure 1-figure supplement 2.pdf]

[acetate]=1mM

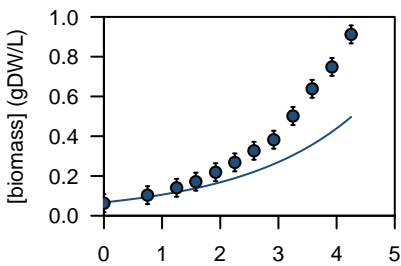

[acetate]=10mM

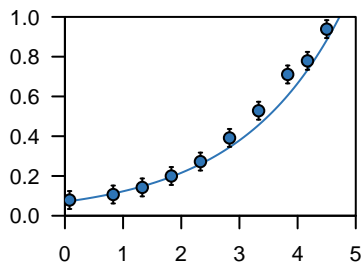

[acetate]=30mM

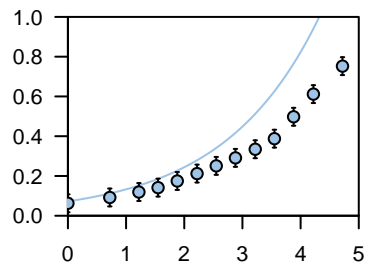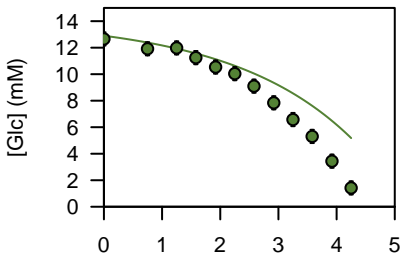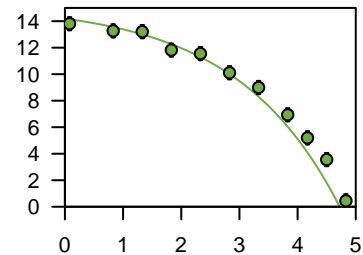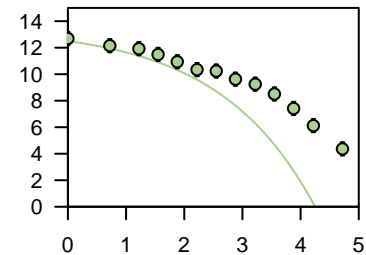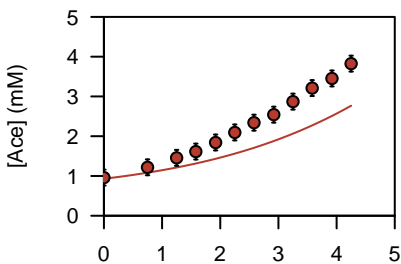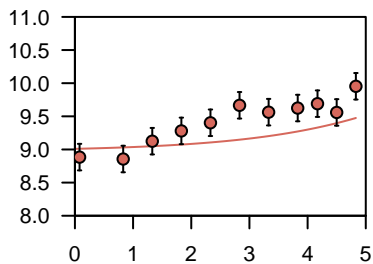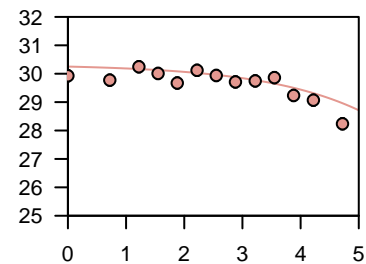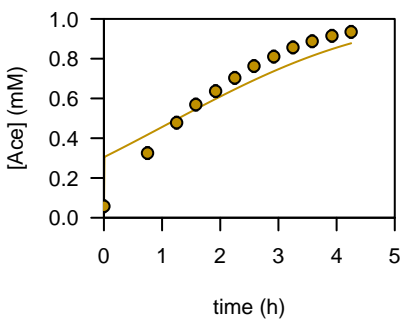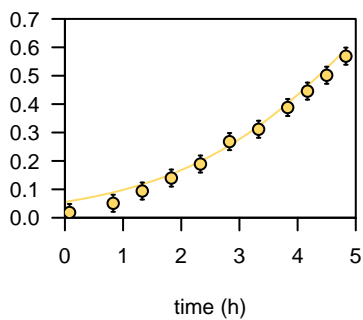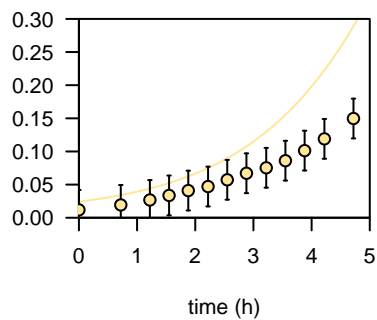

Supplement: Supplementary file 1. [file elife-63661-supp1.zip › acetate_regulation/results/Figure 1-figure supplement 3.pdf]

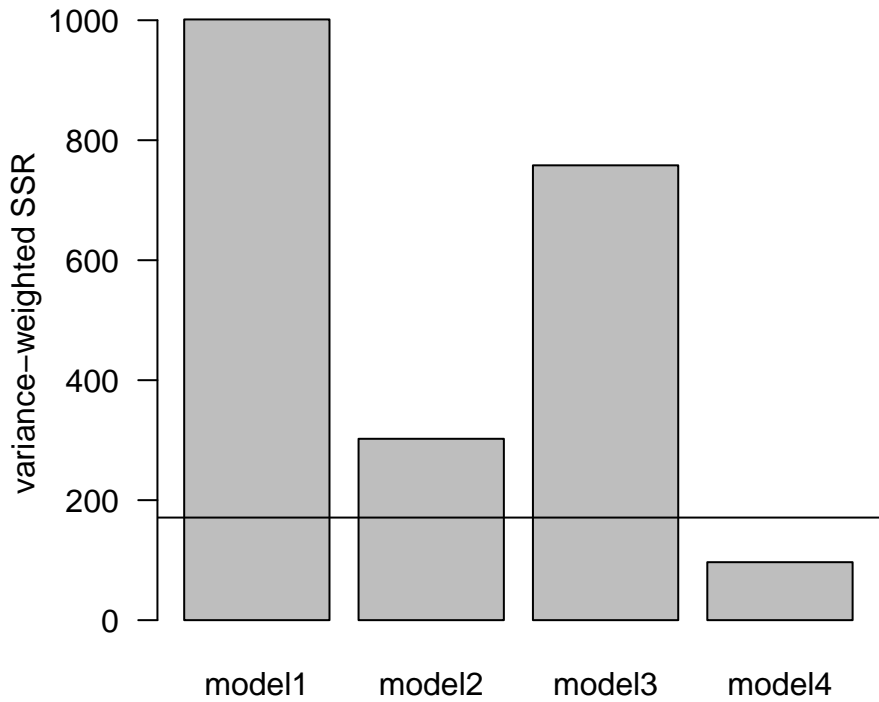

Supplement: Supplementary file 1. [file elife-63661-supp1.zip › acetate_regulation/results/Figure 1B.pdf]

[acetate]=1mM

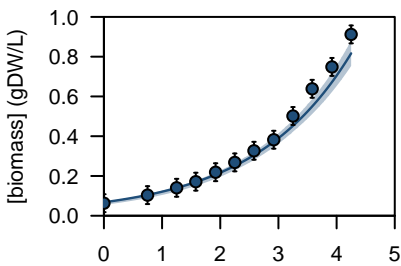

[acetate]=10mM

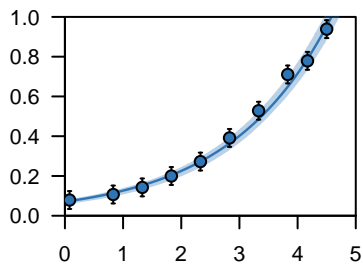

[acetate]=30mM

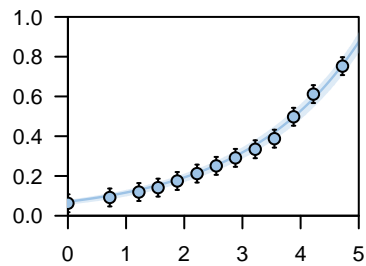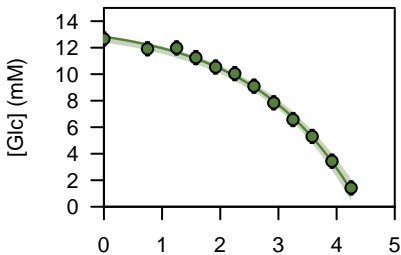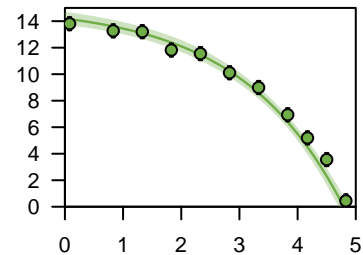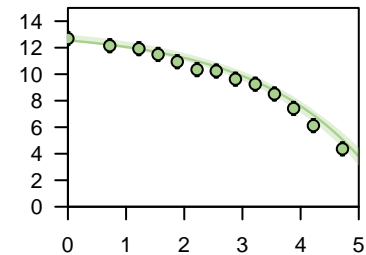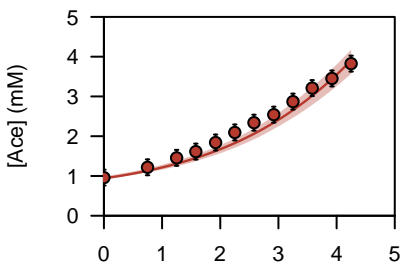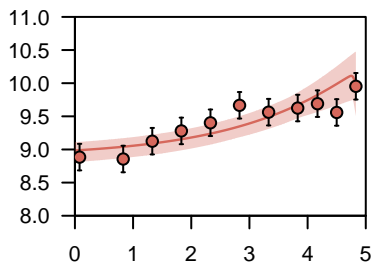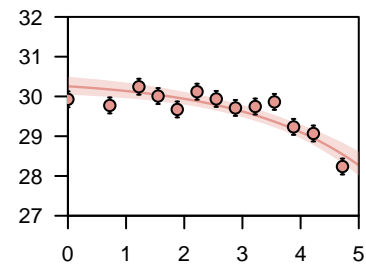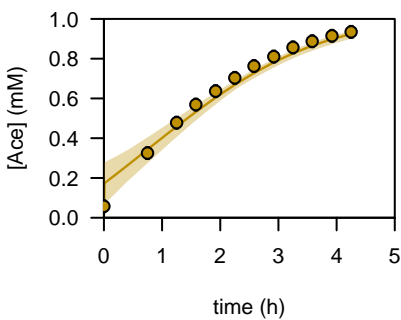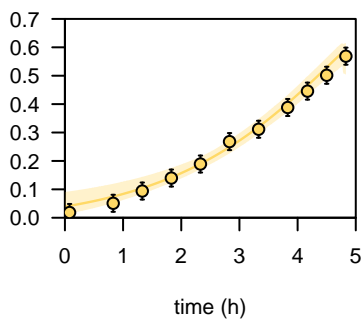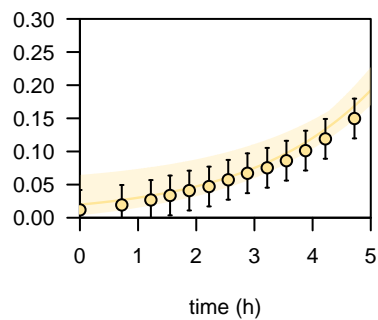

Supplement: Supplementary file 1. [file elife-63661-supp1.zip › acetate_regulation/results/Figure 1C.pdf]

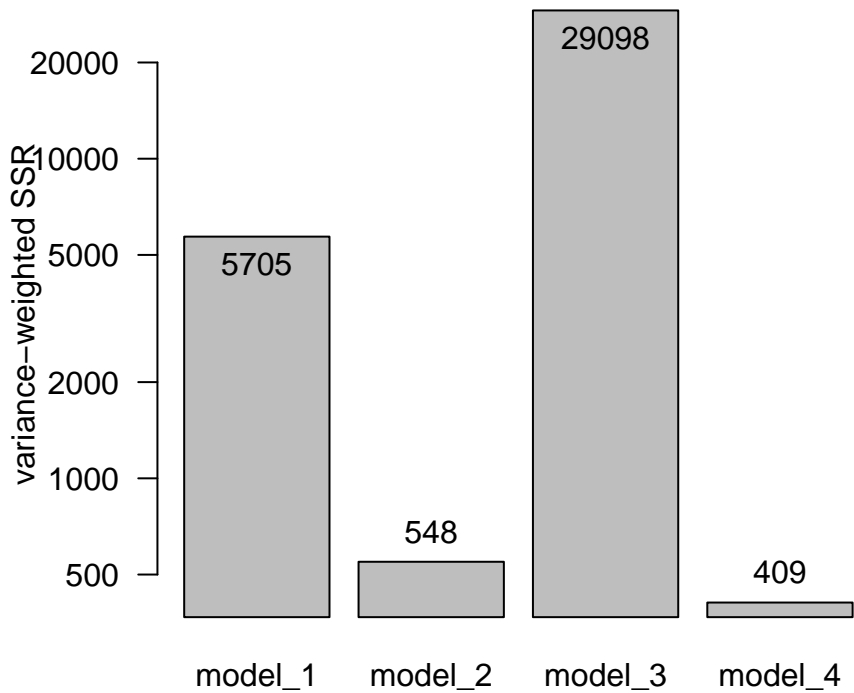

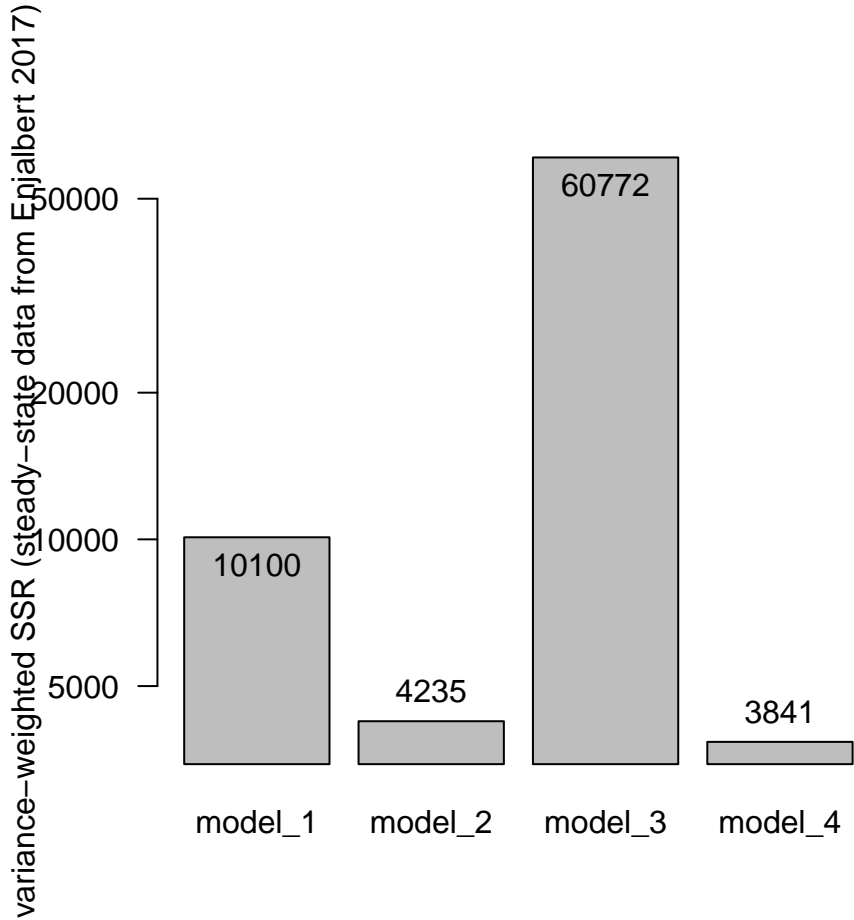

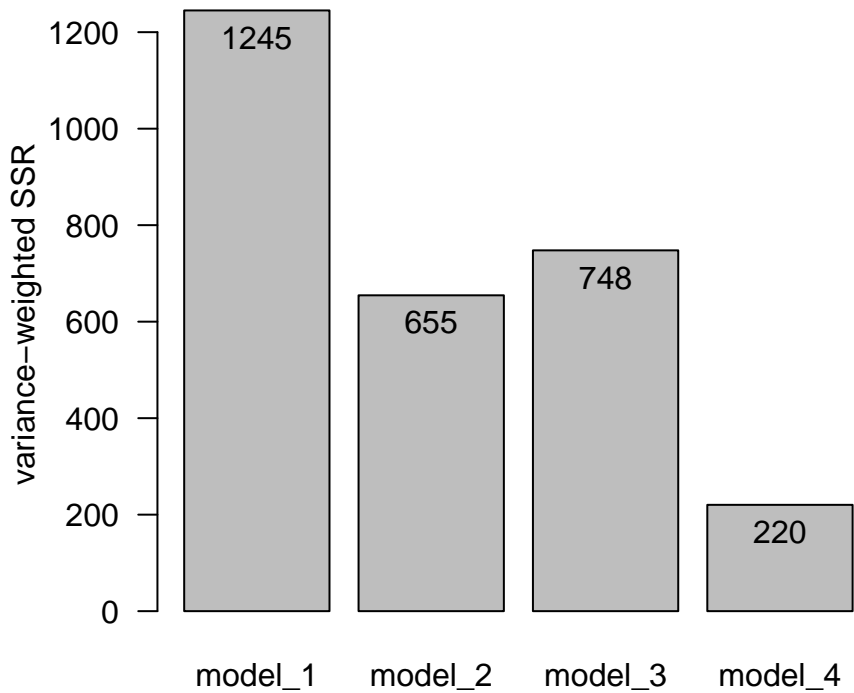

Supplement: Supplementary file 1. [file elife-63661-supp1.zip › acetate_regulation/results/Figure 3–figure supplement 4.pdf]

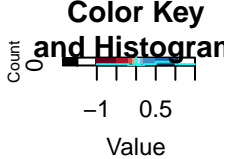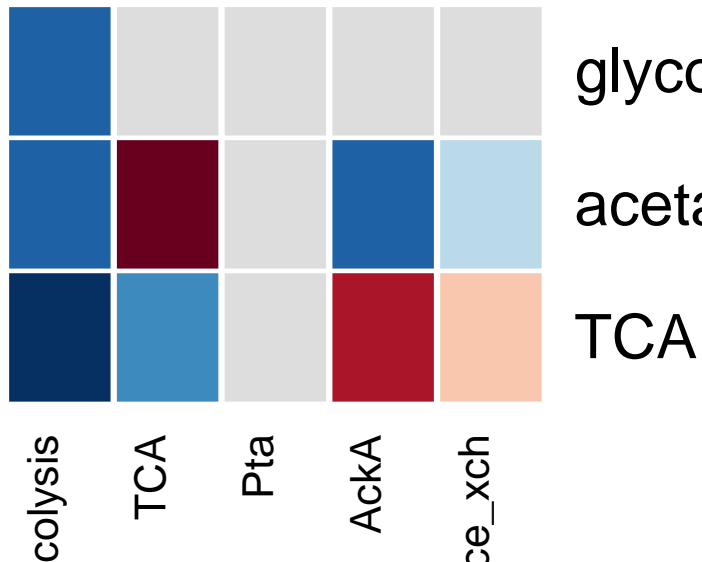

Supplement: Supplementary file 1. [file elife-63661-supp1.zip › acetate_regulation/results/Figure 4.pdf]

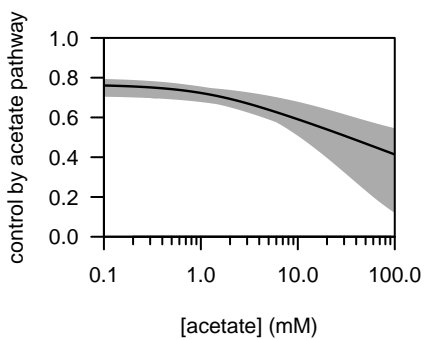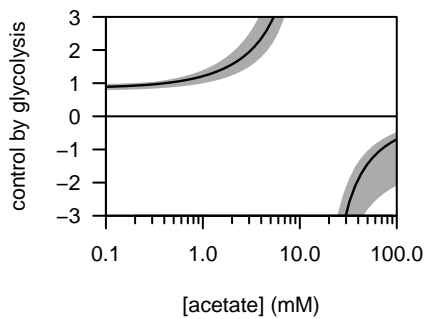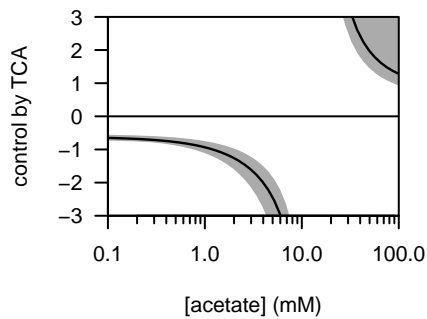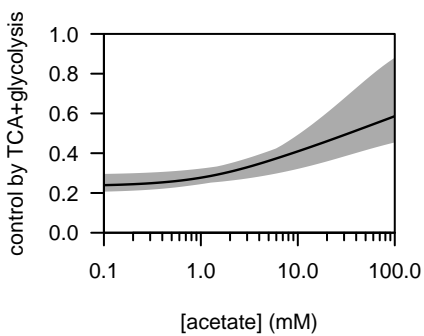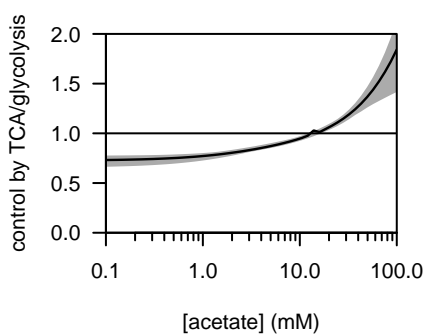

Supplement: Supplementary file 1. [file elife-63661-supp1.zip › acetate_regulation/results/Figure 5.pdf]

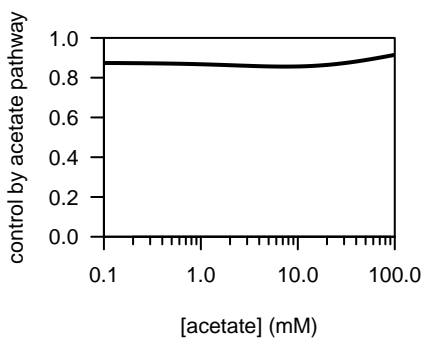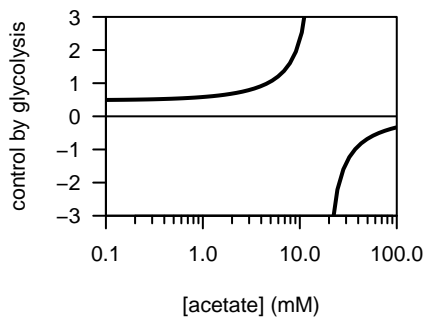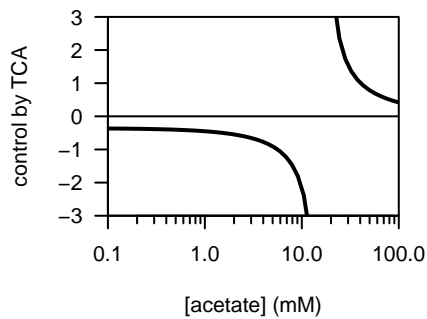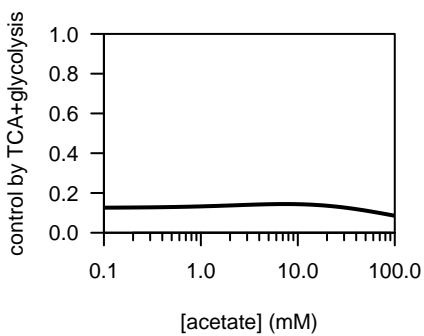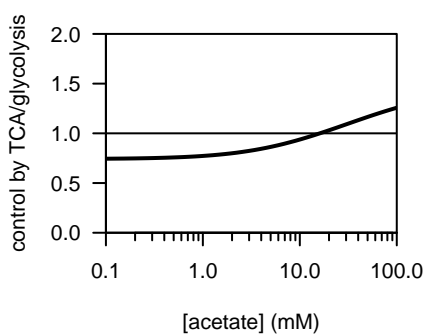

Supplement: Supplementary file 1. [file elife-63661-supp1.zip › acetate_regulation/results/Figure 5–figure supplement 1.pdf]

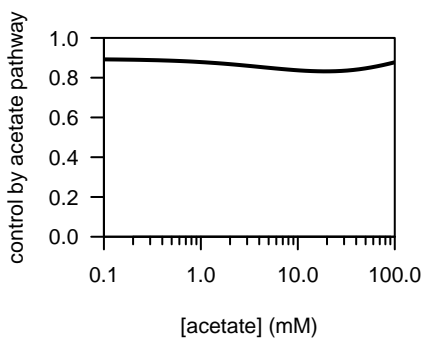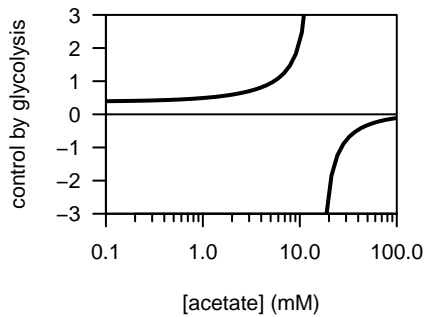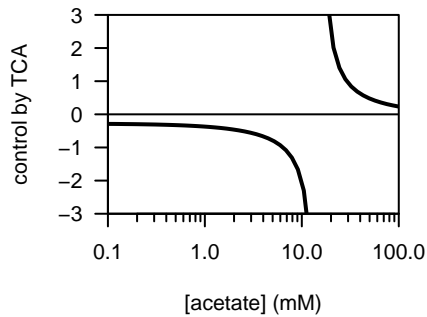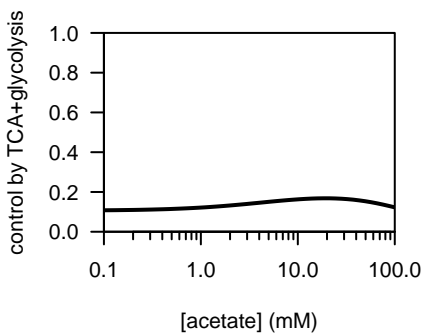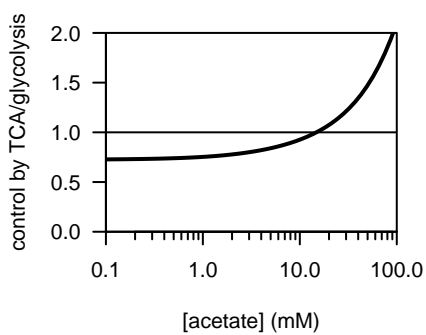

Supplement: Supplementary file 1. [file elife-63661-supp1.zip › acetate_regulation/results/Figure 5–figure supplement 2.pdf]

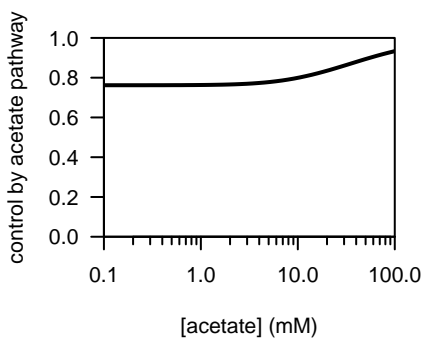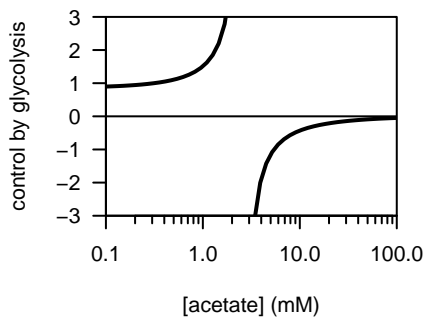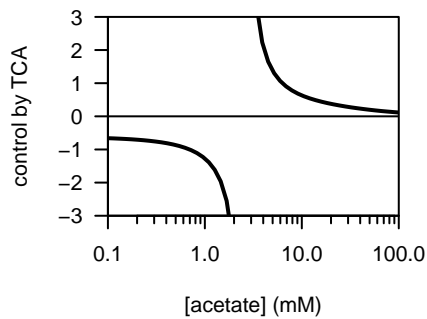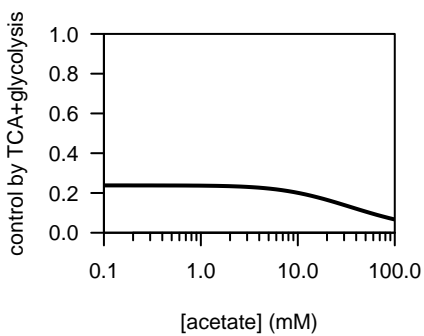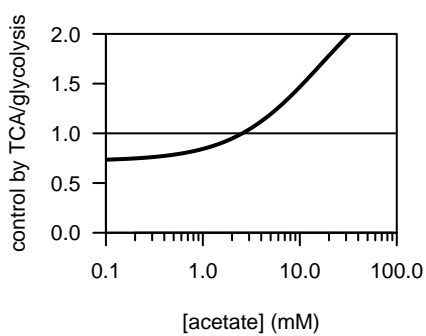

Supplement: Supplementary file 1. [file elife-63661-supp1.zip › acetate_regulation/results/Figure 5–figure supplement 3.pdf]

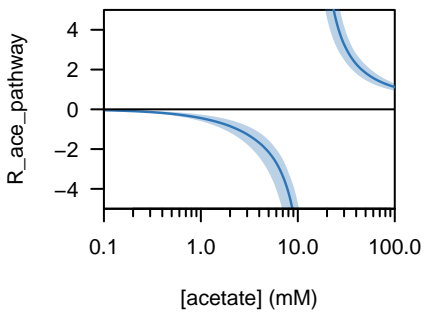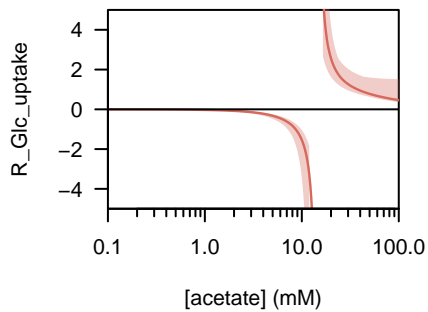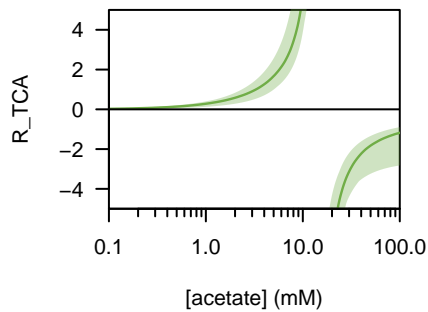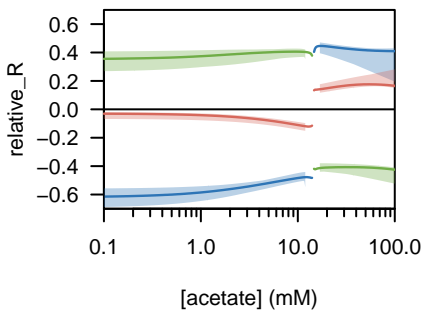

Supplement: Supplementary file 1. [file elife-63661-supp1.zip › acetate_regulation/results/Figure 6.pdf]
