# Supplementary material for: Control and regulation of acetate overflow in Escherichia coli
Source: eLife. 2021 Mar 15;10:e63661. doi: 10.7554/eLife.63661 (PMC8021400; doi:10.7554/eLife.63661)
Supplement: Transparent reporting form [file elife-63661-transrepform.docx]

***eLife’s* transparent reporting form**

We encourage authors to provide detailed information *within their submission* to facilitate the interpretation and replication of experiments. Authors can upload supporting documentation to indicate the use of appropriate reporting guidelines for health-related research (see [EQUATOR Network](http://www.equator-network.org/%20)), life science research (see the [BioSharing Information Resource](https://biosharing.org/)), or the [ARRIVE guidelines](http://www.plosbiology.org/article/info:doi/10.1371/journal.pbio.1000412) for reporting work involving animal research. Where applicable, authors should refer to any relevant reporting standards documents in this form.

If you have any questions, please consult our Journal Policies and/or contact us: [editorial@elifesciences.org](mailto:editorial@elifesciences.org).

**Sample-size estimation**

- You should state whether an appropriate sample size was computed when the study was being designed
- You should state the statistical method of sample size computation and any required assumptions
- If no explicit power analysis was used, you should describe how you decided what sample (replicate) size (number) to use

Please outline where this information can be found within the submission (e.g., sections or figure legends), or explain why this information doesn’t apply to your submission:

This work focuses on bacterial metabolism and does not involve human or animal research, hence we were not limited *per se* in terms of number of samples and replicates. All experiments were carried out in tightly controlled conditions to ensure a high degree of reproducibility. For each of the four conditions analyzed in this work, transcriptomics data were obtained from four independent biological replicates, each with two technical replicates, as stated in the Materials and Methods section (paragraph “Transcriptomics experiments”) and in the legend of Figure 2.

**Replicates**

- You should report how often each experiment was performed
- You should include a definition of biological versus technical replication
- The data obtained should be provided and sufficient information should be provided to indicate the number of independent biological and/or technical replicates
- If you encountered any outliers, you should describe how these were handled
- Criteria for exclusion/inclusion of data should be clearly stated
- High-throughput sequence data should be uploaded before submission, with a private link for reviewers provided (these are available from both GEO and ArrayExpress)

Please outline where this information can be found within the submission (e.g., sections or figure legends), or explain why this information doesn’t apply to your submission:

Transcriptomics data presented in this article were obtained from four independent biological replicates, each with two technical replicates (as detailed in the legend of figure 2 and in the Materials and Methods section). All data were included for analyses, i.e. no data was excluded. The corresponding dataset is made available to the community via the ArrayExpress database (www.ebi.ac.uk/arrayexpress), under accession number E-MTAB-9086, as detailed in the Materials and Methods section of the manuscript (paragraph “Transcriptomics experiments”). We uploaded all raw data as well as detailed protocols for growth experiments, sample collection, sample analysis and data processing.

**Statistical reporting**

- Statistical analysis methods should be described and justified
- Raw data should be presented in figures whenever informative to do so (typically when N per group is less than 10)
- For each experiment, you should identify the statistical tests used, exact values of N, definitions of center, methods of multiple test correction, and dispersion and precision measures (e.g., mean, median, SD, SEM, confidence intervals; and, for the major substantive results, a measure of effect size (e.g., Pearson's r, Cohen's d)
- Report exact p-values wherever possible alongside the summary statistics and 95% confidence intervals. These should be reported for all key questions and not only when the p-value is less than 0.05.

Please outline where this information can be found within the submission (e.g., sections or figure legends), or explain why this information doesn’t apply to your submission:

For transcriptomics experiments, EcoCyc (<https://ecocyc.org>) was used for Gene ontology analyses, as detailed in the Materials and Methods section of the manuscript (paragraph “Transcriptomics experiments”). P-values of gene enrichments were estimated using a Fisher exact test with Bonferroni correction, as detailed in the Materials and methods section.

To assess the goodness-of-fit of each model and determine whether they described the data with sufficient accuracy, we used χ2 statistical tests with parameter α set to 0.95 to define a 95 % confidence threshold, as detailed in the Materials and methods (section “Goodness-of-fit analysis.”). We used a monte carlo analysis (with 500 iterations) to determine the 95% confidence intervals for all model analyses (section “Global sensitivity analysis.”). All the R scripts used to construct and to analyse the models (including χ2 statistical tests and sensitivity analyses) are provided in the Supplementary Information (Supplementary file 1) and at [https://github.com/MetaSys-LISBP/acetate_regulation](https://github.com/MetaSys-LISBP/acetate_regulation/) to ensure reproducibility and reusability.

(For large datasets, or papers with a very large number of statistical tests, you may upload a single table file with tests, Ns, etc., with reference to sections in the manuscript.)

**Group allocation**

- Indicate how samples were allocated into experimental groups (in the case of clinical studies, please specify allocation to treatment method); if randomization was used, please also state if restricted randomization was applied
- Indicate if masking was used during group allocation, data collection and/or data analysis

Please outline where this information can be found within the submission (e.g., sections or figure legends), or explain why this information doesn’t apply to your submission:

This information doesn’t apply to this work (data were not grouped, all data were included in the model).

**Additional data files (“source data”)**

- We encourage you to upload relevant additional data files, such as numerical data that are represented as a graph in a figure, or as a summary table
- Where provided, these should be in the most useful format, and they can be uploaded as “Source data” files linked to a main figure or table
- Include model definition files including the full list of parameters used
- Include code used for data analysis (e.g., R, MatLab)
- Avoid stating that data files are “available upon request”

Please indicate the figures or tables for which source data files have been provided:

All models are provided in SBML and COPASI formats in the Supplementary Information (Supplementary File 1) and at [https://github.com/MetaSys-LISBP/acetate_regulation](https://github.com/MetaSys-LISBP/acetate_regulation/). The calibrated kinetic model has also been deposited in the Biomodels database (<https://www.ebi.ac.uk/biomodels>) with the identifier MODEL2005050001.

All the R scripts used to perform the simulations, to analyse the models and to generate the figures are provided in the Supplementary Information (Supplementary file 1) and at [https://github.com/MetaSys-LISBP/acetate_regulation](https://github.com/MetaSys-LISBP/acetate_regulation/) to ensure reproducibility and reusability. All source data used for model construction and calibration are also provided in the Supplementary Information (Supplementary file 1) and at [https://github.com/MetaSys-LISBP/acetate_regulation](https://github.com/MetaSys-LISBP/acetate_regulation/).
